# Association Study between an SNP in CD147 and Its Expression With Acute Coronary Syndrome in a Jiangsu Chinese Population

**DOI:** 10.1097/MD.0000000000001537

**Published:** 2015-10-23

**Authors:** Jinchuan Yan, Yu Mao, Cuiping Wang, Zhongqun Wang

**Affiliations:** From the Department of Cardiology, Affiliated Hospital of Jiangsu University, Zhenjiang, Jiangsu Province, PR China (JY, YM, CW, ZW).

## Abstract

CD147 is an important molecule in the inflammation and proteolysis process. This molecule crucially contributes to the initial and progression of atherosclerotic lesions. A single nucleotide polymorphism in CD147 gene, the *rs8259* T/A in the 3′-untranslated region, is responsible for its expression in various cells. This study assessed whether the genetic variation *rs8259* is associated with acute coronary syndrome (ACS) and CD147. A total of 943 ACS subjects and 439 stable angina subjects, and 851 controls were genotyped for *rs8259* polymorphism by polymerase chain reaction restriction fragment length polymorphism and DNA-sequencing method. Plasma soluble CD147 (sCD147) level was measured by enzyme-linked immunosorbent assay. CD147 mRNA and protein expression in peripheral blood mononuclear cells were tested by real-time quantitative polymerase chain reaction and western blot, respectively. We found that TT genotype and T-allele frequency of CD147 *rs8259* in ACS patients were much lower than the other patient groups. Significant difference was not observed between stable angina and controls. CD147 T allele was negatively related to ACS. ACS patients exhibited the highest CD147 expression in peripheral blood mononuclear cells and plasma sCD147 level. The plasma sCD147 levels in the culprit vessel were higher than those in the radial artery. In ACS patients, AA gene carriers had the highest CD147 levels, whereas TT gene carriers had the lowest CD147 levels. Linear regression analysis showed that genotypes and disease conditions contributed 49% to the change of the plasma CD147 level. These results suggested that the single nucleotide polymorphism of CD147 gene *rs8259* T/A was associated with ACS susceptibility. Allele T gene may decrease the relative risk of suffering from ACS through downregulation of CD147 expression.

## INTRODUCTION

Acute coronary syndrome (ACS) is a severe subtype of coronary heart disease (CHD); it is a progressive inflammatory disease of the vascular wall and remains among the leading underlying causes of mortality worldwide.^1^ Atherosclerotic plaque rupture is the key mechanism of ACS. Matrix metalloproteinases (MMPs) play an important role in the rupture of the vulnerable plaques by degrading the fibrous of the plaque.^2,3^ Monocytes/macrophages and activated vascular smooth muscle cells in atherosclerotic plaque both can secrete MMPs.^4^ Recent study has suggested that the synthesis of these MMPs is induced by the extracellular MMP inducer (EMMPRIN, R&D Co, Shanghai, China; CD147).^5,6^

CD147 is a 58-kDa cell surface glycoprotein of the immunoglobulin superfamily; it was originally described on the surface of tumor cells, wherein it can induce the synthesis of MMPs in adjacent fibroblasts through homotypic CD147–CD147 interactions.^7,8^ Although the pathogenic mechanisms of CD147 remain unclear, CD147 has recently been identified as a mere marker of inflammation.^9–11^ CD147 possesses a pivotal role in the complex processes of atherogenesis, atheroprogression, and acute atherosclerothrombosis, that is, it is consistently associated with the risk of cardiovascular disease.^6,12–14^ Furthermore, CD147 is important in angiogenesis,^15^ which is a process also found in advanced atherosclerotic plaque and is discussed to promote plaque destabilization. Hence, CD147 may support several impaired pathways of the atherosclerotic plaques, thereby leading to possible rupture of vulnerable plaques; however, the mechanism of CD147 regulation is not clearly elucidated. In this study, we investigated its potential association with ACS and whether the *rs8259* variants in CD147 gene are of functional relevance for CD147 expression.

Various single nucleotide polymorphisms (SNPs) in humans are associated with numerous diseases and conditions. Furthermore, Wu et al^16^ genotyped SNPs at the CD147 locus and found that the 3′-untranslated region (3′-UTR) T/A (*rs8259*) SNP modified the association correlation between CD147 expression and miRNA-492; however, no study has determined the association between SNPs of CD147 and cardiovascular risk, especially ACS. Therefore, we used the SNP *rs8259* in the CD147 3′-UTR locus that has previously been associated with inflammatory in psoriasis. SNP *rs8259* was employed as the genetic marker to discuss the possible association between CD147 variants and incident ACS in a prospective study. Moreover, we investigated whether changes in CD147 expression are functionally relevant for ACS.

## MATERIALS AND METHODS

### Patients and Controls

A total of 1382 CHD cases including 943 patients with ACS and 439 patients with stable angina (SA), and 851 control cases from the Department of Cardiology, Affiliated Hospital of Jiangsu University were enrolled in this study. ACS and SA are defined as our previous reported.^16^ Coronary artery diseases (CADs) in the controls were also excluded by coronary angiography. Patients with tumor, infection, or liver/kidney disease were excluded in Table 1. ACS patients were followed for 6 months for the major adverse cardiac events (MACE). MACE was defined as hospitalization due to cardiovascular causes, recurrent nonfatal myocardial infarction, unplanned Percutaneous Coronary Intervention, new-onset heart failure, and cardiovascular mortality.

**TABLE 1:**
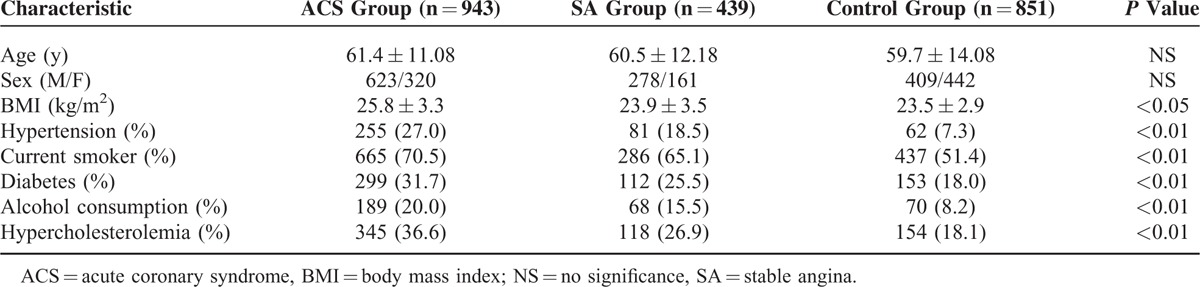
Baseline Characteristics of Study Population

The study plan was reviewed and approved by the ethical review committee, and informed consent from all patients and controls was strictly enforced.

### Blood Sample

Artery blood samples were obtained from the radial artery and culprit vessel. Peripheral blood mononuclear cells (PBMCs) were purified from peripheral blood by standard density-gradient centrifugation using lymphocyte separation medium (LTS1077 TBD Science, Tianjin, China). In brief, blood was centrifuged at 500 g for 20 minutes at room temperature. After centrifugation, the top plasma layer was collected and stored at −80 °C. The precipitation was then diluted 1 : 1 with sterile phosphate buffered saline before layering over the separation medium. PBMCs were carefully aspirated with spinning at 400 g for 20 minutes. The cells were then washed 3 times and used for reverse-transcriptase polymerase chain reaction (RT-PCR) or western blot.

### Genetic Analysis

Total genomic DNA was extracted from peripheral venous blood with phenol–chloroform extraction. The polymorphism of *rs8259* T/A was detected by PCR-restriction fragment length polymorphism method. The primer sequences (forward: 5′-GAGTCCACTCCCAGTGCTTG-3′; reverse: 5′-CTCGTGAAACACTTCAGAAGGAAAAGA-3′) were used as previously reported.^17,18^ In a thermal cycler (Bio-Rad Co, Shanghai, China), 0.2 μg genomic DNA was used for PCR amplification. The restriction enzyme MboI (MBI Co, Shanghai, China) digested the 162 bp PCR products into 2 fragments: 137 and 25 bp for the *rs8259* A allele, whereas the uncut PCR products (162 bp) represent T allele. Subsequently, the digested products were analyzed in 3% agarose gels and stained with ethidium bromide. Several individual DNA fragments were selected for sequencing analysis (Invitrogen, Shanghai, China).

### Real-Time RT-PCR

Total RNA was extracted from PBMC with TRIzol (Invitrogen) following the manufacturer's instructions. A quantitative real-time RT-PCR was performed with a Bio-Rad thermocycler and an SYBR green kit (Bio-Rad) according to the recommendations of the manufacturer. Each sample was run in triplicate and in 3 independent experiments. The relative CD147 mRNA expression, which was normalized to the level of comparing different groups, was determined by 2^−▿▿ct^ method. The following sense and antisense primers were used: for CD147 sense: 5′-CAGAGTGAAGGCTGTGAAGTCG-3′; antisense: 5′-TGCGAGGAACTCACGAAGAA-3′; and for GAPDH (endogenous control): 5′-CTGCACCACCAACTGCTTAG-3′ (sense); 5′-AGGTCCACCACTGACACGTT-3′ (antisense).

### Western Blot

PBMCs were lysed with RIPA buffer (Cell Signaling, Shanghai, China) that supplemented with a protease inhibitor cocktail (Sigma, Shanghai, China). BCA Protein Assay Kit, Thermo Scientific Shanghai, China was used to detect the protein concentrations. After separating with SDS-polyacrylamide gel electrophoresis, proteins were transferred onto a polyvinylidene difluoride membrane. The membranes were immunoblotted with antibodies against CD147 (Proteintech, Huhan, China) or β-actin (Sigma), followed by a horseradish peroxidase-conjugated secondary antibody. Immunoreactivity was detected with ECL (Pierce, Shanghai China).

### Enzyme-Linked Immunosorbent Assay

The plasma level of CD147 was determined by enzyme-linked immunosorbent assay as the protocol of Human EMMPRIN Immunoassay Quantikine kit (R&D Co). A microplate reader (Bio-Rad Co) was used to determine the optical density of each well. The intra-assay and inter-assay coefficients of variation were 3% and 5%, respectively.

### Statistical Analysis

Statistical graphs were performed with Graph pad software (Prism 5.0, Shanghai, China). The enumeration data are presented as mean ± standard deviation and compared by analysis of variance. Cross-tabulation and standard v2 test were used to compare the frequency distribution of genotypes. The relative risk was assessed by odds ratios (OR, with 95% confidence intervals, CI). The effect level of the SNP polymorphism to the plasma CD147 in radial artery was analyzed by linear regression method. *P* < 0.05 was considered statistically significant.

## RESULTS

### CD147 Gene 3′-UTR Region *rs8259* Polymorphism Analysis

**FIGURE 1:**
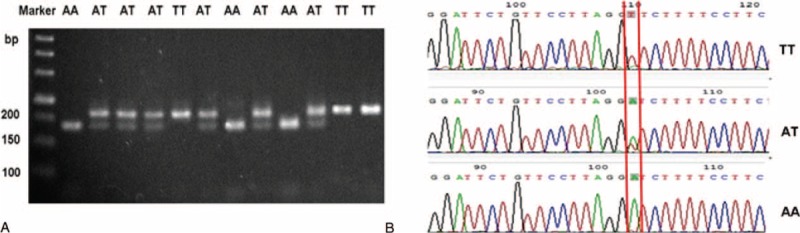
CD147 gene 3′-untranslated region *rs8259* polymorphism analysis. A, The polymerase chain reaction product is 162 bp in length (including 137 bp and 25 bp). B, Different genotypes of sequencing results.

The PCR product is 162 base pair in length and was digested by MboI. The uncut PCR product (162 bp) representing the T allele was separated from the fragments (137 and 25 bp). This separation resulted from the digestion of the A allele by electrophoreses in a 2.5% agarose gel (Figure 1A). The result was identified by sequencing. The sequencing results are shown in Figure 1B.

### Association of CD147 *rs8259* T/A Polymorphism With ACS Susceptibility and Prognosis

Table 1 shows the prevalence and distribution of clinical and lifestyle factors in 3-group members. As expected, ACS and SA subjects exhibited increased occurrence of several known cardiovascular risk factors (body mass index, hypertension, hyperlipidemia, alcohol consumption, smoking, and diabetes) compared with the control members and the ACS patients have the highest occurrence. The distribution of alleles is consistent with the Hardy–Weinberg equilibrium (P > 0.05). As shown in Tables 2 and 3, the frequencies of the T allele and the TT genotype in ACS group were significantly lower than those in SA and control groups (*P* < 0.01, Table 3). Significant difference was not observed between the SA and control groups (*P* = 0.737 and 0.238). The T allele decreased the risk of ACS compared with the SA group (OR = 0.824 95% CI: 0.736–0.921) and the control group (OR = 0.808 95% CI: 0.736–0.888). No significant differences in the genotypes and allele frequencies were found between the SA and control groups.

**TABLE 2:**
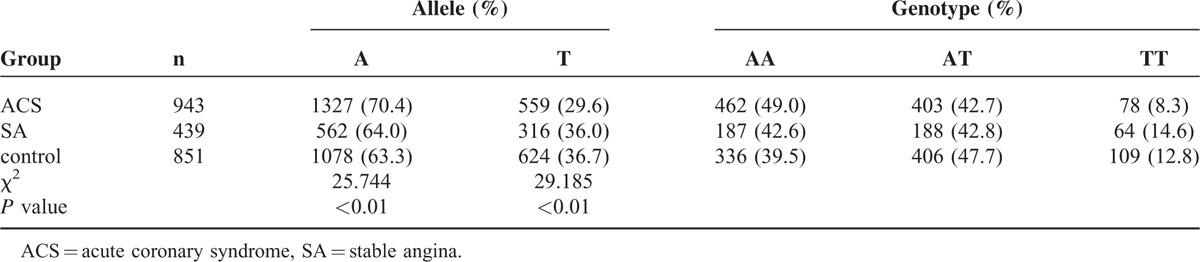
Distribution of CD147 Gene Polymorphism in the 3 Groups

**TABLE 3:**
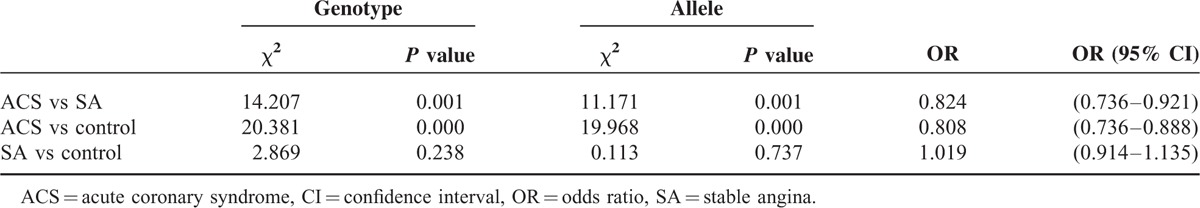
The Differences in Each Group (Partitions of χ^2^)

**FIGURE 2:**
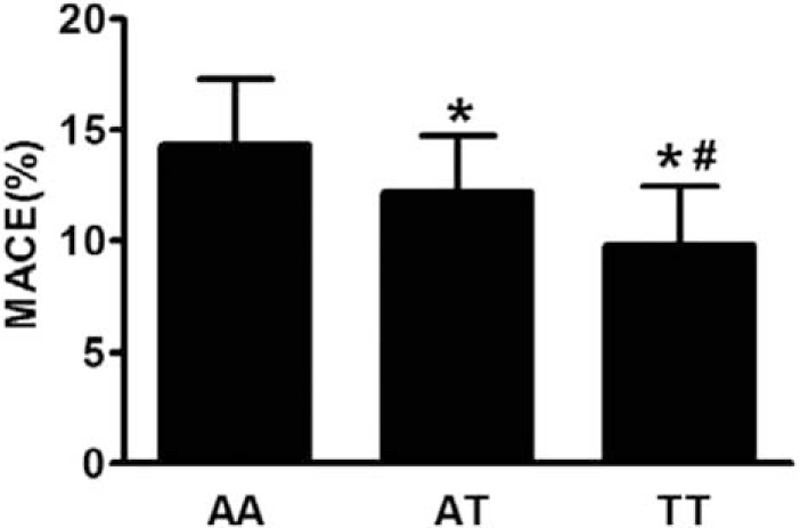
The rs8259: T/A genotype affected the prognosis of acute coronary syndrome patients. Data represented mean ± standard deviation, ^∗^vs AA genetype, *P* < 0.05; ^#^ vs AT genetype, *P* < 0.05. MACE = major adverse cardiac events.

In ACS patients, we found that patients with T-allele carrier were along with lower rate of MACE (Figure 2).

### Increased PBMC CD147 Expression and Plasma sCD147 in ACS Patients

**FIGURE 3:**
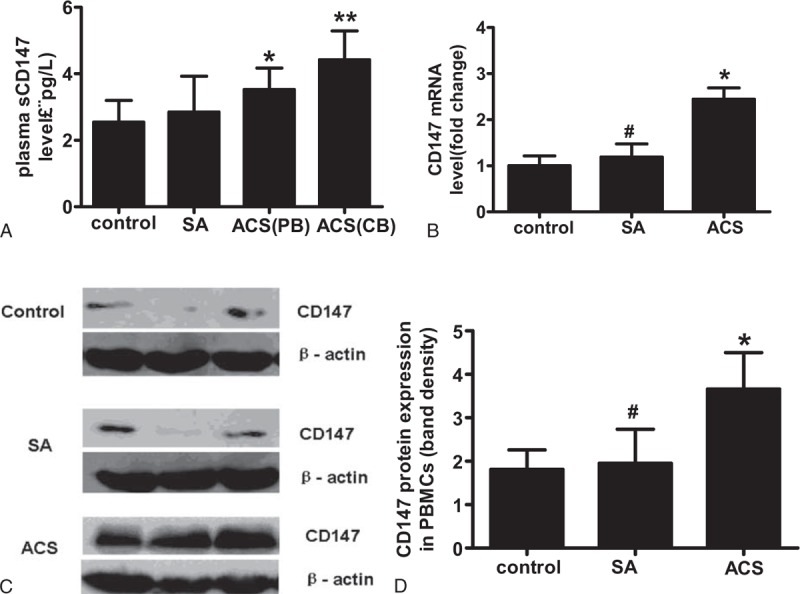
Comparison of CD147 mRNA, protein level in PBMCs and plasma level of CD147 from ACS patients, SA patients, and healthy controls. A, Enzyme-linked immunosorbent assay for CD147 plasma level. B, Reverse-transcriptase-quantitative polymerase chain reaction for CD147 mRNA expression in PBMCs. C, Western blotting for CD147 protein level in PBMCs. Representative relative CD147 protein expression was detected by western blotting analysis. D, Ratio of density of CD147 with β-actin quantified with BandScan5.0 software. Data were mean values ± SD from independent samples analyzed in 3 replicates, *P* < 0.05. All data were mean values ± SD from independent samples analyzed in 3 replicates, ^∗^vs control, *P* < 0.01;^ ∗∗^*P* < 0.05 vs ACS(PB); ^#^vs ACS, *P* < 0.01. ACS = acute coronary syndrome, CB = coronary blood, PB = peripheral blood, PBMCs =  peripheral blood mononuclear cells, SA = stable angina, SD = standard deviation.

To investigate the possible contribution of CD147 to ACS, we quantified and compared the CD147 expression in PBMCs, and plasma soluble CD147 (sCD147) (from the radial artery and culprit vessel) in patients. Figure 3 shows that CD147 mRNA and protein levels in PBMCs and plasma sCD147 levels in ACS patients were significantly higher than those in SA patients and controls. In ACS patients, the plasma sCD147 levels from the culprit artery were higher than those from the radial artery. No significant difference was observed between the control and SA groups.

### Association of CD147 *rs8259* T/A Polymorphism With CD147 Expression

**FIGURE 4:**
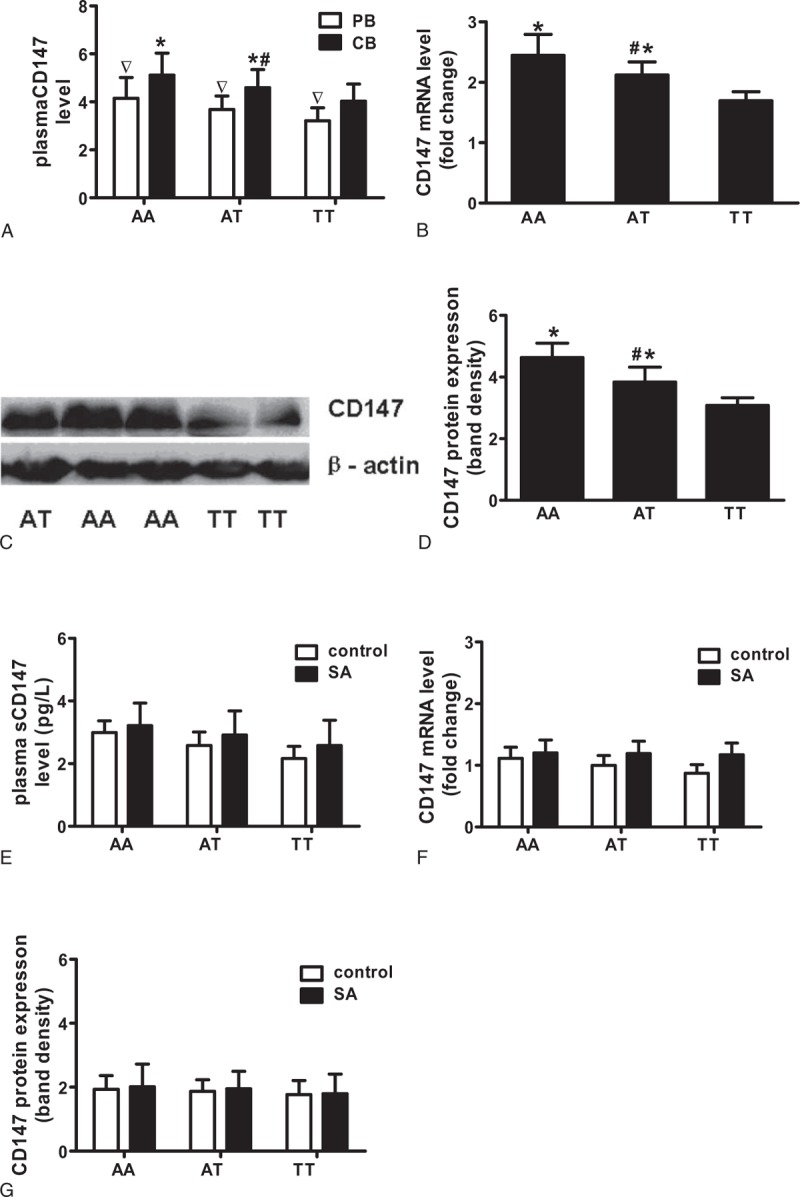
The *rs8259*: T/A genotypes affected CD147 expression in PBMCs and plasma sCD147 levels from ACS patients with different genotypes. A, Enzyme-linked immunosorbent assay analysis of relative plasma sCD147 level in ACS patients. B, Real-time polymerase chain reaction analysis of relative CD147 mRNA expression in PBMCs from ACS patients. C, Representative relative CD147 protein expression was detected by western blotting analysis in PBMC from ACS patients. D, Ratio of density of CD147 with β-actin quantified with BandScan5.0 software for ACS patients. E, F, G, Plasma sCD147 levels, PBMC CD147 mRNA and protein levels in SA group and controls. Data represented mean ± SD, ^∗^vs TT genotype, *P* < 0.05; ^#^vs AA genotype, *P* < 0.05, ^▿^vs CB, *P* < 0.05. ACS = acute coronary syndrome, CB = coronary blood, PB = peripheral blood, PBMCs = peripheral blood mononuclear cells, sCD147 = soluble CD147, SA = stable angina, SD = standard deviation.

To investigate whether the *rs8259* T allele contributes to the lower level of CD147 expression and lower ACS incident rate, we further analyzed the CD147 expression in PBMC (mRNA and protein) and sCD147 in plasma with different *rs8259* genotypes in ACS patients. Data showed that CD147 levels (mRNA, protein in PBMCs, and molecule in plasma) were all expressed at the lowest levels in TT genotype (Figure 4A–D). Although SA patients and control group have the lower CD147 expression and have no significant difference among 3 genotype subgroups, TT genotype subgroup had the lowest CD147 expression in PBMC and sCD147 levels in plasma (Figure 4E–G).

### Effect of the *rs8259* Polymorphism on Plasma CD147 Level

The plasma CD147 level was set as dependent variable. Genotype, sex, age, body mass index, blood pressure, blood lipid level, smoking, diabetes, and presence or absence of ACS was used as related factors. Multiple linear regression method was employed to analyze the influence of CD147 gene polymorphism on the plasma levels of CD147. The result showed that the regression equation was statistically significant (*F* = 29.841, *P* < 0.01), that is, genotype and classification of diseases (ACS and no ACS groups) were linearly correlated with the plasma levels of CD147 (*P* < 0.01). They were independent factors that affected the plasma levels of CD147. The adjusted determination coefficient *R*^2^ value was 0.490, which suggests that the genotypes (AA, AT, and TT) and the disease types (ACS and no ACS) contributed 49% to the change of the plasma CD147 level. The relative result is shown in Table 4.

**TABLE 4:**
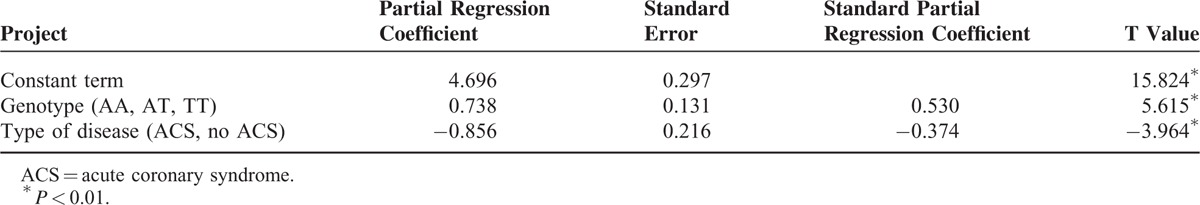
Multivariate Linear Regression Coefficient Estimation and Test Results of the Effect of CD147 Genotype and ACS on Plasma Level of CD147

## DISCUSSION

To our knowledge, this study is the first to investigate the association of CD147 polymorphism and ACS susceptibility. Several novel findings were obtained as follows. First, CD147 expression in PBMCs and levels of plasma sCD147 were upregulated in ACS patients compared with SA patients and controls. In ACS patients, plasma sCD147 levels in the culprit vessel were higher than those in the radial artery. Second, the carriers of the T allele for the SNP *rs8259* that occurred in the 3′-UTR of the CD147 gene were associated with decreased ACS susceptibility and lower rate of MACE after ACS. Third, T-allele carriers in ACS patients had lower CD147 expression, whereas A-allele carriers had higher CD147 expression. Multiple linear regression analysis found that the genotypes (AA, AT, and TT) and the disease types (ACS and no ACS) contributed 49% to the change of the plasma sCD147 level.

CD147, a member of the immunoglobulin super family, is a well-known potent inducer of extracellular MMPs. Recent studies have demonstrated that CD147 plays important roles in lymphocyte development and immune response.^19^ CD147 expression was found to be increased in inflammatory disease, such as rheumatoid arthritis,^20–24^ systemic lupus erythematosus,^25^ ischemic injury,^26,27^ and atherosclerosis.^28–31^ In CAD patients, higher levels of CD147 expression were observed not only in plasma, but also on platelets, monocytes, and granulocytes in circulation. Meanwhile, higher levels of CD147 on platelets appear to correlate with more severe situation of CAD.^32^ The present research found that ACS patients exhibited higher CD147 levels not only in PBMCs, but also in plasma than SA patients and controls. In addition, the higher levels of plasma sCD147 are found in the culprit vessel. These results indicate that CD147 was more relevant to unstable plaque and acute vascular disease.

SNPs in human immune gene (HLA-G) show that allele frequencies vary between asthma patients and controls.^33^ Wu et al^17^ found that the SNP *rs8259* in the CD147 3′-UTR locus SNPs is associated with psoriasis susceptibility. T allele decreases the risk of psoriasis suffering. In this study, we investigated the relationship between *rs8259* A/T of CD147 gene polymorphism and ACS. The results showed that carriers of the *rs8259* T allele showed decreased risk for ACS. Association was not observed between the SNP and SA. This finding may imply that the *rs8259* gene polymorphism is directly linked to atheromatous plaque vulnerability rather than the initiation and development of atherosclerosis. Interesting, we also found that the T-allele carrier decreased the rate of MACE. The mechanism needs to be further explored.

SNPs at the 3′-UTR may affect gene expression at the post-transcriptional level by interfering protein binding, polyadenylation, or miRNA binding.^34^ Research found that the *rs8259* T/A gene polymorphism in 3′-UTR of CD147 alter gene expression through affecting miRNA targeting activity. In the present study, we detected CD147 expression in PBMCs and plasma sCD147 levels and analyzed them with the genotypes. Results showed that the levels of plasma sCD147 and PBMC CD147 expression in the T-allele carriers were significantly lower than those in the A-allele carriers. In each group, the CD147 expression in subjects with the TT or AT genotype was lower than that with the AA genotype. We hypothesize that the change from SNP A to T leads to the decreased CD147 protein expression, which affects atherosclerotic disease progression and plaque destabilization. We also found that genotype and classification of diseases (ACS and no ACS) were linearly correlated with the plasma levels of CD147 through multiple linear regression analysis. The result supported that the SNP alters CD147 expression to affect ACS susceptibility.

Basing on these findings, we concluded that the polymorphism *rs8259* T/A in 3′-UTR of CD147 was significantly associated with ACS susceptibility, but not with SA. Further study is warranted to investigate the contribution of CD147 gene variation to ACS susceptibility. Notably, our data also indicated that the polymorphism *rs8259* T/A may alter the level of CD147 expression and secretion, thus affecting the risk on suffering from ACS. Investigating the underlying mechanism on how the variation in CD147 contributes to its expression and the mechanism on how to irritate the development of ACS is interesting. Considering the frequency of the detrimental genotype, the results also may be of public health concern.

## LIMITATIONS

There are several limitations in the present study. First, we did not analyze the relationship between the genotype and angiography lesions, it may better reveal the relationship between the genotype and vulnerability plaque. Second, our study was performed in a relatively small number of patients and limited in Asian population in Jiangsu Province of China. Further studies with larger cohorts of patients should be performed to illustrate the correlation of the CD147 gene polymorphism with ACS susceptibility. Third, a larger number of genes and SNPs are related with ACS, studies independently or in combination with other CD147 SNPs and other genes are needed. In additional, human disease is affected by gene–environment interaction; a large number of factors could influence results.
